# Reinforcement of Stearic Acid Treated Egg Shell Particles in Epoxy Thermosets: Structural, Thermal, and Mechanical Characterization

**DOI:** 10.3390/ma11101872

**Published:** 2018-10-01

**Authors:** Ahmer Hussain Shah, Yuqi Zhang, Xiaodong Xu, Abdul Qadeer Dayo, Xiao Li, Shuo Wang, Wenbin Liu

**Affiliations:** 1 Key Laboratory of Superlight Material and Surface Technology, Ministry of Education, College of Materials Science and Chemical Engineering, Harbin Engineering University, Harbin 150001, China; ahmer.shah@buitms.edu.pk (A.H.S.); zhangyuqi7@hrbeu.edu.cn (Y.Z.); abdul.qadeer@buitms.edu.pk (A.Q.D.); shawn@hrbeu.edu.cn (X.L.); wangshuo123@hrbeu.edu.cn (S.W.); liuwenbin@hrbeu.edu.cn (W.L.); 2 Department of Textile Engineering, Balochistan University of Information Technology, Engineering and Management Sciences, Quetta 87300, Pakistan; 3 Department of Chemical Engineering, Balochistan University of Information Technology, Engineering and Management Sciences, Quetta 87300, Pakistan

**Keywords:** egg shell, stearic acid, modification, particle characterization, epoxy composites, dynamic mechanical analysis, adhesion effectiveness

## Abstract

This work reports the modification of egg shell (ES) particles by using stearic acid (SA) and their reinforcement in the epoxy matrix. The ES treatment via SA was optimized, the optimum conditions for concentration, temperature, and time were found to be 2.5%, 85 °C, and 50 min, respectively. The untreated ES (UES) and treated ES (TES) particles were characterized by Fourier transform infrared spectroscopy (FTIR), differential scanning calorimetry (DSC), X-ray diffraction (XRD), scanning electron microscope (SEM), particle size distribution, and contact angle. FTIR confirmed the chemical modification of SA on ES surface and DSC reflects an endothermic peak at 240 °C. XRD reveal a decrease in crystal size and crystallinity, while contact angle increases to 169° from 42°. The SEM observations clearly reflect a distinct decrease and separation of small domains of ES particles thus improving an increased surface area. Afterwards, the UES and TES particles were reinforced in epoxy at 15 and 20 weight (wt.) % loading. The tensile tests confirmed a 22% increase in elongation as compared to pure epoxy due to the hydrogen bonding between TES particles and matrix. The lowest brittleness was recorded for TES/epoxy composites on 20 wt % loading. The TGA confirmed the improved thermal stabilities at 20 wt % loading of TES particles in matrix, the improvements in *T*_5%_, *T*_10%_, and *T*_20%_ values were recorded as 33, 26, and 21 °C higher than the corresponding values for neat matrix. The TES/epoxy composites on 20 wt % showed 41% increase in storage modulus as compared to the pristine epoxy, and cross-link density reaches to 2.71 × 10^−3^ from 1.29 × 10^−3^ mol/cm^3^ for neat matrix. The decline in *tan δ* height and improvement in *T_g_* were also observed. The best adhesion effectiveness was recorded for TES/epoxy composites. This simple and economical modification technique can enhance the application of ES particles in various polymeric coating and composites applications.

## 1. Introduction

In the modern world, scientists and process industries are focusing on the use of biomaterials, either as raw materials or as a phase reinforcement to improve the properties of material. Epoxies—having various structures depending on the hardener used—are cheap materials and widely used in number of industries. Aliphatic and aromatic amine are commonly used hardeners for epoxies curing, the aliphatic amine type hardeners are cheap and can cure epoxy at room temperature, but their properties are lower as compared to the aromatic amine type hardeners. However, both hardeners showed the similar behavior for brittleness, the high brittleness is considered as major associated drawback [1]. The polymer structure has poor resistance to crack propagation and low impact strength along with low thermal stability. One way to overcome the toughness of the thermosets like epoxies is to reinforce a material either in fiber or particle forms both at macro and nano levels [2,3,4,5]. The reinforcement phase physically interacts and/or chemically reacts with epoxy or hardener depending on the treatment [6].

The egg shell (ES) is a bio-waste generated in enormous quantity, as eggs are part of daily meal around the world, the chemical composition include 95% calcite embedded with an organic matrix [7]. China is in the first place for the production and consumption of eggs having 40% share in world consumption [8]. Table 1 shows the composition of ES particles [9]. The ES particles have been used in different applications after various treatments [10,11,12,13,14,15]. The carbonized ES particles (at 500 °C for 3 h) reinforced metal matrix composites show increased tensile strength, hardness and fatigue strength on 12.5 wt % ES loading but toughness was reduced [16]. The micro size ES particles reinforced epoxy composites showed a 72% improvement in toughness at 5 wt % ES loading reinforced composites. However, on higher amount (>5 wt %) the toughness decreases and reaches to the pure material [17]. Reduction in toughness was the result of agglomeration of particles at higher loading amount. The nano ES particles epoxy composites on 1 to 5 wt % ES were studied by Mohan and Kanny, the best results for the tensile and impact strength were observed on 2 wt % ES reinforcement, while on higher loading the decline was observed in the properties values due to serious agglomeration [18]. In the nano composites, the aliphatic room temperature hardener was used and lower amount of ES was loaded, but the results were much better than the micro size particle composite [16,17]. However, the associated cost for the size decrease and heat treatment of particles are not in favor for commercial application.

As the particles size decreases, the particles agglomeration at higher loading increases. The agglomeration can be avoided or reduced by effective surface treatment with suitable modifying agents, which improve the dispersion of fillers in matrix [19]. The calcium carbonate (CC) is the major component of ES particles, and an easy and effective surface modifications technique for calcium carbonate by reacting with stearic acid (SA) is reported [20,21]. SA is a cheap organic acid having hydrophilic head and oleophobic tail [22]. Several reports are reported for increased performance of composites by reinforcing CC treated with SA. Lam et al. [23] reported that SA treatment of CC improved the dispersion and adhesion of polypropylene/CC composites, which improved the mechanical and thermal stability of composites. In another study by Osman et al. [24], it was found that the amount necessary to gain high level of dispersion depends on the amount of SA coating. Monolayer coating of CC particles achieves more beneficial effects as compared to bilayer formation, if the amount of SA increases on surface. From literature analysis it is believed that SA treatment of ES particles may be adopted as a good technique to improve reinforcing affects in composites. At this stage, it is therefore necessary to optimize the process of ES treatment such that to prevent the formation of bilayer on ES surface. We believe that this method can effectively modify the ES particles’ surface, and ultimate results of modification can enhance the reinforcement effects [25].

In this work, we report a simple and economical modification method for the ES particles by using stearic acid (SA), the treatment parameters were optimized. The efficacy of treatment was studied by FTIR, XRD, DSC, SEM, and water contact angle. The untreated and SA treated ES particles were reinforced in epoxy at higher loading i.e., 15 and 20 wt %, and their impacts on the tensile, thermal, and thermomechanical properties were evaluated along with structural changes by FTIR analysis.

## 2. Materials and Methods

### 2.1. Materials

ES were purchased from the local food market at Harbin, China. Epoxy (diglycidylether of bisphenol-A (DGEBA), E-51) having 184–195 g/eq EEW (epoxy equivalent weight), triethylenetetramine (TETA), stearic acid (99%, molecular weight 284.48), and dimethylbenzene (xylene) were procured from Sigma–Aldrich, Shanghai, China. Sodium hydroxide (NaOH) was purchased from Sigma–Aldrich with a 99.99% purity grade. Ethanol and deionized water were used throughout the study.

### 2.2. Treatment

The inner membrane of ES was manually separated; afterwards, shells were boiled in water for 30 min and overnight oven dried at 80 °C. The ES particles size was reduced by grinding in a home grinder and particles passed 500 mesh were used in the study. Later, the impurities from particles were removed by 30 min soaking in ethanol at 15:1 liquor ratio, ultrasonicated and stirred. After that, ES particles (100 g) were dipped in a SA solution i.e., water 500 mL + 250 mL of 0.014 mol NaOH and 0–3% varied concentration of SA, mixture was stirred at 300 rpm at various temperatures (25 to 105 °C at 20 °C ramp) and time periods (20 to 60 min at 10 min interval). The particles were separated and overnight vacuum dried at 80 °C.

### 2.3. Process Optimization

The process optimization of modification of ES particles was carried out by studying four parameters—i.e., sediment volume, oil-wet coefficient (*O_w_*), active ratio % (*A_d_*), and viscosity ratio (*V_R_*). The procedures are defined below:

1. 1.0 g of ES sample was placed in a cylinder containing 30 mL of absolute ethanol with a stopper at the top. The cylinder was shaken well up and down for 3 min and kept at room temperature for 15 min. Settling volume *V_set_* (mL/g) was calculated as per
  (1) $$V_{set}=\frac{V_{sed}}{m}$$
  where *V_sed_* is the sediment volume in mL, *m* is the mass of sample in grams, and *V_set_* is volume per gram of sediment.
2. 0.2 g of the ES particles were taken and poured into a beaker containing 50 mL of deionized water. A measured quantity of methanol (*V*) was added drop wise to make the surface particles setting down. Oil-wet coefficient (*O_w_*) was calculated by using the equation
  (2) $$O_{w}=\frac{V}{V+50}\times 100$$
3. Fixed amount of ES powder was placed in a separatory funnel to which 200 mL of water was added and shaken manually for 1 min. The mixture was kept still for 20–30 min so that the stratification of ES particles becomes obvious. After clear stratification of layers, the settled ES particles were separated from the layered. The layered particles were carefully taken and kept at 105 °C in vacuum oven to constant weight (≤0.1 mg). Active ratio (*A_d_*) was calculated by the equation [26].
  (3) $$A_{d}=\frac{M_{f}}{M_{t}}\times 100$$
  where *A_d_* is the active ratio %, *M_f_* is mass of floating part, and *M_t_* is total mass of the sample.
4. 10% ES particles solution with distilled water was flowed through a viscometer at atmospheric pressure and temperature, similar was repeated for pristine distilled water. The ratio of 10% ES particles solution viscosity to pure water viscosity was taken as viscosity ratio (*V_R_*).

After the process optimization, following characterization methods were used to distinguish between untreated egg shell (UES) and treated egg shell (TES) particles.

### 2.4. Composite Fabrication

50 g of E51 resin was taken in a glass beaker and heated at 80 °C; the 15 or 20 wt % of UES or TES particles were added in the beaker and vigorously stirred at 300 rpm for 30 min by using a mechanical mixer. Later, for the better dispersion of particles in resin, the mixture was ultrasonicated for 30 min, and cooled down to room temperature. The stoichiometric amount of TETA hardener was added in the mixture and stirred for further 10 min. The mixture was poured into the desired testing sample dimension moulds. The samples were cured at room temperature for 24 h and post cured at 90 °C for 2 h.

### 2.5. Measurements and Analyses

Fourier transform infrared (FTIR) spectrum analysis was applied to study the chemical structure of UES and TES particles and epoxy composites by casting a thin film. A small amount of sample powder was mixed with KBr and film was prepared and examined on PerkinElmer Spectrum 100 spectrometer (Waltham, MA, USA). The spectra were recorded at 4 cm^−1^ resolution in the range of 4000–400 cm^−1^.

X-ray diffraction (XRD) measurements were conducted on an X’Pert High Score PW3209 diffractometer for the investigation of crystal structure and other impurities. The tests were performed at room temperature from 20° to 80° of 2*θ*. The measurements were further used to analyze UES and TES crystal sizes by using Scherrer equation (Equation (4)). The samples were prepared by uniformly spreading the powder on a quartz sample holder.

(4) $$L=\frac{K\;\lambda }{\beta \;\cos\;\theta }$$

where *L* is the crystallite size, *λ* is the wavelength of X-ray in nanometers (nm) which is taken as monochromatized CuKα radiation = 0.154184 nm, *K* is crystallite shape constant (0.9), *β* is diffraction peak profile at half maximum height in radians, and *θ* is the dominant peak of 2*θ* of material in radians.

Differential scanning calorimetry (DSC) measurements were performed on differential scanning calorimeter model TA Q200, TA Instruments, New Castle, DE, USA, under 50 mL/min steady flow rate of nitrogen. The 5.0 mg of sample was carefully weighed in a DSC sample pan at room temperature. The experiments were conducted from 50 to 300 °C at 20 °C/min heating rate.

Morphology and surface characteristics were investigated via scanning electron microscope (SEM) (CamScan SU 8010, Oxford Instruments, Oxford, UK) at 20 kV. The samples for SEM testing were collected carefully and sealed in glass bottles. The SEM copper plate was covered by conductive resin tape and particles/composites were distributed on the tape and gold coated.

Contact angle measurement of UES and TES particles was evaluated by sessile drop method. A thin pallet of both the powders was made by compression and dropping water droplet on it. The test was measured at 25 °C. The particle sizes were calculated on Malvern ZETASIZER nano series, UK, by dispersing them in a water media at 25 °C temperature.

Thermogravimetric analysis (TGA) was performed on TA Instruments Q50 at a heating rate of 20 °C/min from 40 to 800 °C under a constant (50 mL/min) nitrogen flow rate.

Tensile tests of the composites were performed on Universal Instron 4467, Instron, Norwood, MA, USA, machine at a cross head speed of 2 mm/min and the load cell capacity of 30.0 kN as per standard ASTM D-683. Dumbbell shape specimens were cast in a mold containing small amount of silicon oil for easy removal of samples after cure. The working gauge length of specimen was 12 mm. A total of five specimens were tested for each category and the average values are reported.

Thermomechanical properties of the composites were estimated under bending mode testing on a dynamic mechanical analyzer (DMA) model Q800 from TA Instruments, USA. 30 × 10 × 2 mm^3^ polished specimens were loaded in a single cantilever mode, and examined under a nitrogen atmosphere from 50 to 150 °C at 3 °C/min heating rate. The sinusoidal strain was applied with 1 Hz frequency by air.

## 3. Results

### 3.1. Optimization of SA Treatment

The effects of concentration of SA, treatment temperature, and time on the ES particles were optimized by evaluating the sediment volume, oil-wet coefficient (*O_w_*), active ratio % (*A_d_*), and viscosity ratio (*V_R_*). The results are summarized in Table 2.

*V_set_* is an important parameter to measure the dispersion of particles in a liquid. If the particles have good dispersion, they have large volume and will not aggregate and rate of settling will be slow. On the contrary, if the powder has poor dispersion, the sedimentation rate will be faster with lower volume. A direct relation was seen between *V_set_* and optimized parameters—i.e., concentration, temperature, and time. It is however sure that an equilibrium volume is reached after a certain time [27], therefore the effect was seen for all process conditions at 15 min time interval. The porosity and surface roughness change along with hydrophobicity of ES particles must have a direct influence on changing the *O_w_* value. In order to find out the effects of modification parameters, the increase of *O_w_* value reflects more attachment of SA on ES particles. While after reaching a certain quantity of concentration, the high dipole–dipole interaction of molecules decreases the surface attachment because of higher molecular motion which negatively influences the surface attachment. Surface treatment of ES particles also changes the active ratio % in liquid. *A_d_* was calculated to see the behavior of particles behavior after modification. Due to the difference in surface tension, well treated, and modified ES floats while poorly interacted particles sink in water. It is reported that before the formation of bilayer on surface, the particles have increasing trend of this ratio which becomes constant after reaching a certain value [26]. The surface becomes hydrophobic as a result of increase in active ratio % due to uniform arrangement of alkyl chains on ES surface. Similar hydrophobic behavior of the ES particles were observed by He and coworkers during the preparation of super hydrophobic material from ES for the oil/water separation [28]. Treatment of particles also affects the viscosity of liquid due to change of surface energy and change of particles size. Well treated ES particles disperse well and hence reduce the viscosity of the solution. The effect of different parameters also shows a difference in *V_R_* values but the difference is small.

The optimized conditions from the obtained results were found to be a 2.5% concentration, 85 °C treatment temperature, and 50 min treatment time. At these processing conditions, the TES particles have highest *V_set_*, *O_w_*, *A_d_*, and lowest *V_R_* values.

#### 3.1.1. Structural Characteristics by FTIR

**Figure 1:**
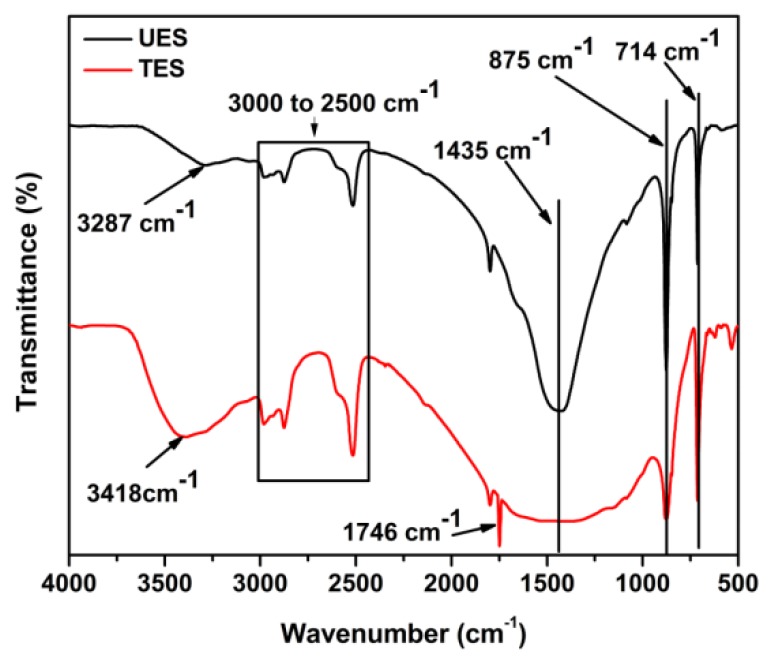
FTIR spectra of UES and TES particles.

**Scheme 1:**
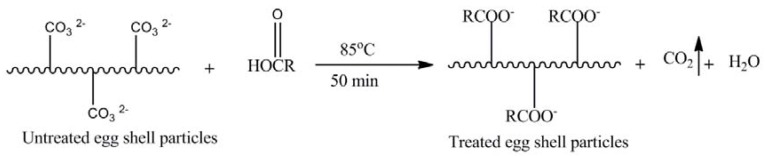
Chemical reaction of SA treatment of ES particles.

The chemical structure before and after treatment of ES particles was examined by FTIR analysis as depicted in Figure 1. It can be seen that UES particles represent characteristic peaks at 1435, 875, and 714 cm^−1^, representing the asymmetric stretch, out-of-plane bend and in-plane bending vibrations of CO_3_^2−^ ion, respectively [29,30]. The absorption bands situated in between 3000 to 2500 cm^−1^ were identified as organic matter and a broad absorption band at 3287 cm^−1^ was due to the stretching vibration of structural H_2_O [31]. The peak positioned at 2505 cm^−1^ can be assigned to acidic hydrogen group –OH stretching [32]. The carbonyl (C=O) stretching vibrations of SA can be observed in the range of 1800 to 1700 cm^−1^. After treatment with SA, it is evident that asymmetric stretch peak of CO_3_^2−^ almost vanishes and out-of-plane bend CO_3_^2−^ decreases in intensity to a greater extent, indicating CO_3_^2−^ on the surface of UES particles react with SA. A clear peak is also observed at 1746 cm^−1^ for C=O stretching in TES which is absent in UES particles, representing the presence of SA ion RCOO^―^. On the other hand, the stretch vibration of structural water of UES was broadened and shifted from 3287 cm^−1^ to 3418 cm^−1^ in TES; which shows a considerable change of structural water in TES particles. The peak of acidic hydrogen group (–OH) stretching also increases in TES which can be attributed to chemical reaction of SA with ES particles. Based on these observations, the proposed reaction is shown in Scheme 1.

#### 3.1.2. XRD

**Figure 2:**
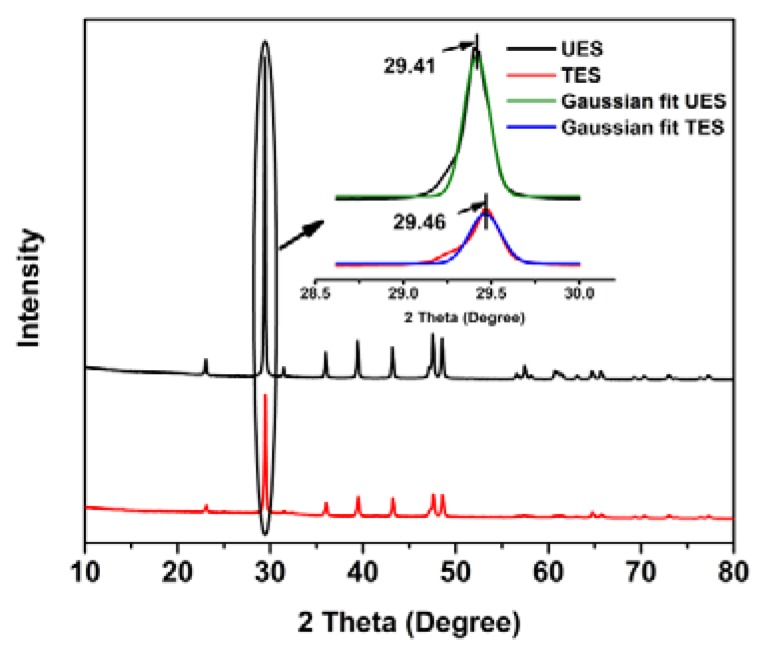
XRD of UES and TES particles.

The XRD of UES and TES particles are illustrated as Figure 2. The UES particles exhibit characteristic peaks at 2*θ* regions of 23, 29, and 35–48, which is consistent with the crystalline nature of calcite of ES particles in the literature [33]. These peaks represent the high ordered crystalline phase of calcite in ES particles. Comparing the XRD of UES and TES particles, we can easily find that the characteristics diffraction peaks are at same positions, which confirmed that after modification new phases were not generated. This represents that only TES surface was modified without affecting the internal crystalline structure. However, it can be observed easily that the relative peaks intensities decreased, which confirms the decrease of crystallinity after the SA treatment. The decrease of crystallinity can be attributed to the covering of ES surface with SA modifier [34]. These XRD results are in good agreement with FTIR results.

The effects of SA treatment on the crystallite size were evaluated by using Scherrer equation (Equation (4)) and shown in Table 3. The dominant peak plot was fitted according to Gaussian function.

We can easily observe the decrease in crystallite size after SA treatment, which suggests the increased surface area for TES particles. The reason could be attributed to the removal of surface impurities during the treatment, as SA removes the proteins and organic matter without affecting the calcite phase. This is important observation for the TES particles application as a calcium carbonate mineral in various drugs, medicated implants or polymers with improved absorption, dissolution, flow ability, solubility, and bio-availability.

#### 3.1.3. DSC

**Figure 3:**
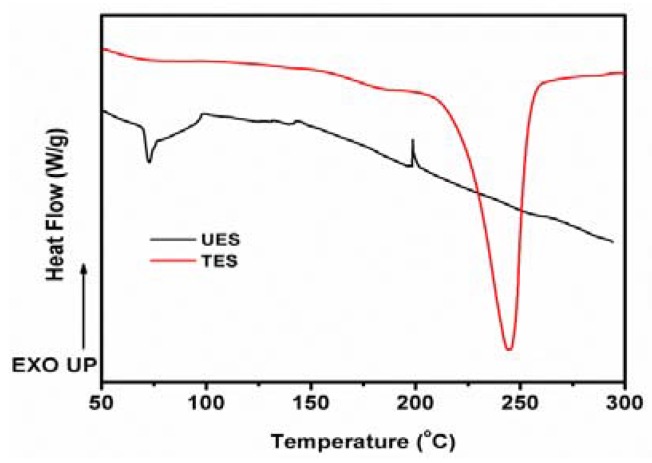
DSC curves of UES and TES particles.

The DSC was employed for the detection of phase transformation of ES particles on heating; plots are produced as Figure 3. For UES particles, few peaks around 60 to 150 °C were detected; these can be related to free water and bio-impurities degradation. For TES particles, an obvious endothermic peak was detected at 240 °C. The endothermic reactions occurred due to volatilization of the molecules, whereas exothermic reaction occurred due to the formation of charring (solid residue) [35,36]. From this analysis, it can be apparent that the surface of TES was modified by chemical bonding of SA to the UES surface to a greater extent.

#### 3.1.4. Morphology

**Figure 4:**
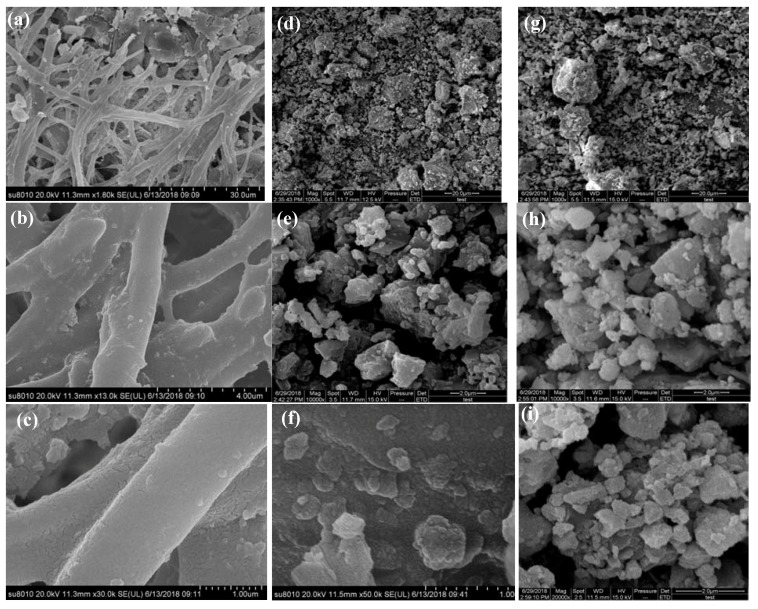
Morphological images of unwashed (**a**–**c**), untreated (**d**–**f**), and treated (**g**–**i**) egg shell particles.

The SEM images of unwashed egg shell, UES, and TES particles at three different magnifications were studied to evaluate the morphological changes on the treatment, SEM are shown as Figure 4.

The gummy net structure having an approximately 1 µm diameter confirms the appearance of proteins, collagen, and sulfated polysaccharides materials in unwashed ES sample, Figure 4a–c, and it is not possible to use unwashed ES as filler in polymer industry. After ethanol washing the UES particles, Figure 4d–f, sufficient removal of proteins and impurities was observed, and gummy material was removed to a good extent and definite irregular shaped particles were observed. On higher magnification, Figure 4e,f, the effect of ethanol washing was clearly visible; however, the particles were agglomerated in bunches even after washing. After the SA treatment, Figure 4g–i, the ES particles agglomerates were dispersed and size was reduced to a greater extent. On high magnification Figure 4h,i, the morphology clearly reflects the breaking of particles with improved edges which means that SA removed the gummy texture and impurities to a greater extent and changed the surface roughness. This improved roughness has a prime importance in TES use in polymers which improve the adhesion of particles with matrix, and ultimately improve the properties of composites.

#### 3.1.5. Contact Angle

**Figure 5:**
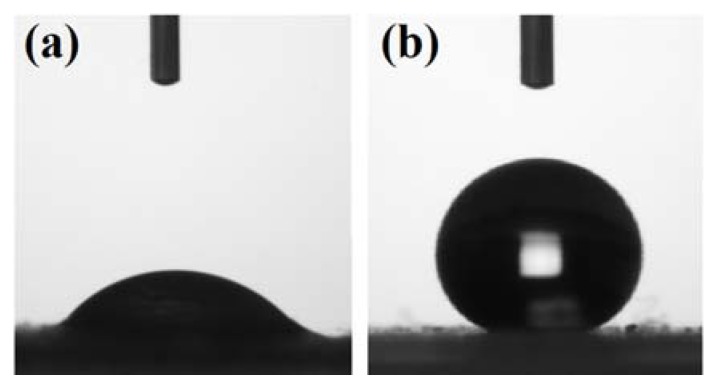
Contact angle of UES (**a**) and TES (**b**).

The hydrophobic characteristics of the ES surface are measured by the contact angle in Figure 5. Water dropping onto UES (Figure 5a) resulted in no firm shape, and water was distributed into the pellet more quickly. The contact angle of water on UES particles was very small (42°) representing a hydrophilic nature of UES particles. The contact angle of TES particles was 169° (Figure 5b), which indicates a very strong hydrophobic surface of the TES particles due to SA modification [37].

#### 3.1.6. Particle Size and Distribution

**Figure 6:**
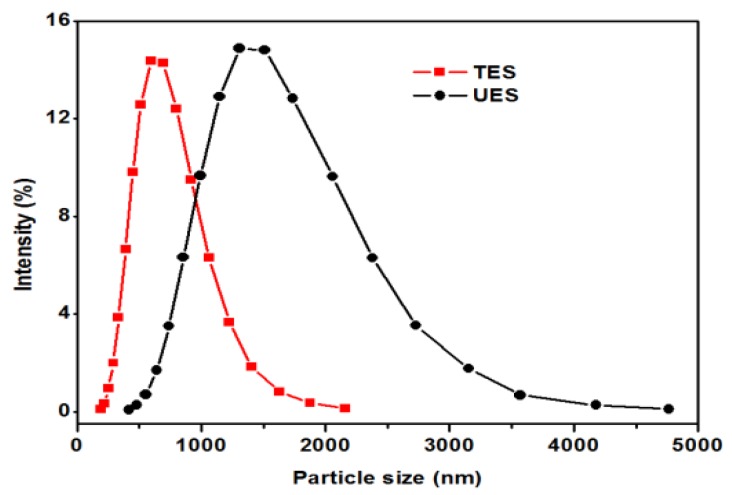
Particles size distribution of UES and TES.

The UES and TES particle sizes after dispersing them in water were calculated at 25 °C temperature. The relative intensities were fitted by using the Guassian fit are shown in Figure 6 and corresponding data is represented in Table 4. It is evident from the plot that UES particles have broader distribution range (500 to 5000 nm) and larger mean particle size (1521 nm). In comparison with UES particles, the TES particles have significantly narrower distribution after SA treatment. The distribution range and mean particle size was recorded at 200–2000 nm and 683 nm, respectively. It is evident from the produced particle size results that SA treatment removed the bio organic impurities to a greater extent and reduces the particle size which is in good agreement with SEM analysis. Moreover, the polydispersity index (PDI) calculated from the plot data also showed decline in the values, which confirms the improved size distribution due to SA reaction on surface causing uniform particle size.

### 3.2. Effects of UES and TES on Epoxy Filled Composites

The epoxy/UES and epoxy/TES composites were prepared on 15 and 20 wt % loading of particles. The effects of particles loading on the epoxy matrix were studied by evaluating the chemical structure, thermomechanical, mechanical, and thermal properties.

#### 3.2.1. FTIR

**Figure 7:**
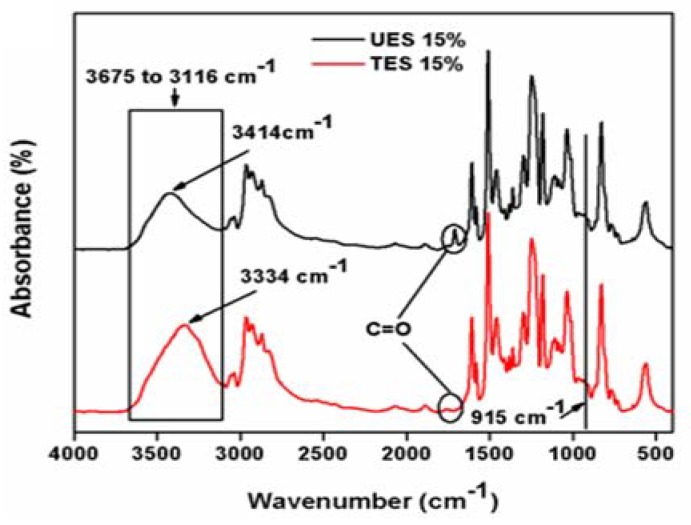
The FTIR spectra of 15% UES and 15% TES filled epoxy composites.

The chemical structure of UES and TES filled epoxy composites were examined by FTIR and plotted as Figure 7.

**Scheme 2:**
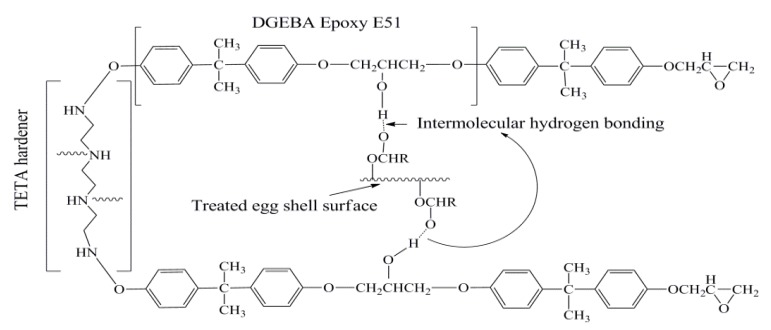
Chemical structure of epoxy with TES particles.

It is important to highlight that there are two main parameters to describe the changes in the chemical structure for UES and TES particles reinforced composites; i.e., shifting in wavelength and intensity variation. The wavelength shift represents either hydrogenation or dehydrogenation if shifted to lower or higher wavelength, respectively [38]. The intensity variation represents the bonds formation or elimination [39]. The spectra of both composites represent characteristics peaks of epoxy. The oxirane group peak located at 915 cm^−1^ corresponds to C–O stretching. The absence of this peak represents that composites were completely cured at given curing conditions. The –OH stretching vibrations are located in the region at 3675 to 3116 cm^−1^. As the reaction progresses, this region between 3675 to 3116 cm^−1^ develops in the structure. The carbonyls stretch (C=O) due to ES particles can be seen at 1710 cm^−1^ and (–OH) peak at 3414 cm^−1^ in UES composites. In TES composites (–OH), the stretching peak is shifted to lower wave number 3334 cm^−1^ while no peak is detected for carbonyl (C=O) stretch. The absence of carbonyl peak reflects the dissociation of formed surface carbonyls with epoxy and is also reported [40]. The area of hydroxyl (–OH) region intensity is also broadened for TES composites. This observation clearly reflects that TES particles are attached with increased hydrogen bonding with epoxy due to greater functionality of TES particles. The proposed chemical structure is shown in Scheme 2.

#### 3.2.2. Particles–Matrix Interaction

**Figure 8:**
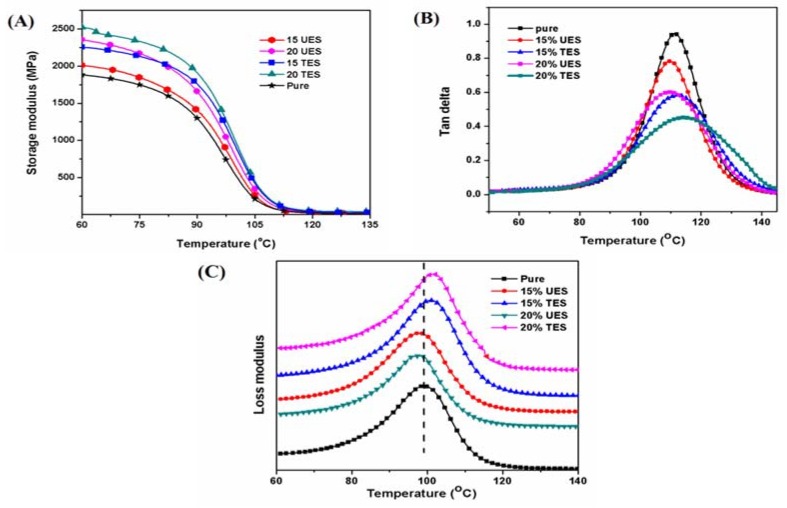
Storage modulus (**A**), *tan* *δ* (**B**) and loss modulus (**C**) of pure epoxy, UES and TES filled epoxy composites.

The interaction of UES and TES particles with epoxy matrix was examined by studying the variation in storage modulus (*E*′), loss modulus (*E″*), and damping factor (*tan δ*) with respect to temperature on a dynamic mechanical analysis (DMA). The plots for particles filled epoxy composites are shown in Figure 8 and the values are tabulated in Table 5.

The study of *E*′, Figure 8A, shows that the *E*′ values steady fall along with temperature increase. Two distinct regions, glassy and rubbery, were observed in all composites. In the glassy region, the high values of *E*′ are due to highly immobile, close and tightly packed matrix components. The UES and TES particles filled composite has increased values of *E*′ values in comparison to neat epoxy due to stiffer nature of particles. In the rubbery region, the 15 wt % UES particles filled composites showed a little higher modulus value due to the interaction of protein contained by the UES particles with epoxy, and they formed a chemically cross-link with epoxy [41]. The TES particles filled epoxy composites showed higher stiffness (*E*′ at 50 °C) values, the stiffness for 15 and 20 wt % composite were 23 and 25% higher than the neat matrix value, respectively. In addition to this, a distinct increase in the rubbery region was also observed. This can be dedicated to the increased porosity, sufficient removal of bio-organic impurities, and reduced TES particles size after SA treatment, along with the chemical cross-linking of alkyl chains with epoxy. This behavior also confirms the improved cross-link points of TES particles in epoxy. The cross-link density was calculated by following equation [42] and values are already summarized in Table 5.

(5) $$\rho =E^{\prime }/3RT$$

where *E*′ is the storage modulus in rubbery plateau (i.e., *T_g_* + 30 °C), *R* is the universal gas constant (8.314472 J/K mol), and *T* (K) is the temperature in a rubbery plateau.

The TES particles filled composite exhibit higher number of cross-link density confirming the improved stiffness of the composite. We can observe that, at 15 wt % UES loading the cross-link density of composite was improved than the neat epoxy, but on 20 wt % UES loading the cross-link density value decreases to a greater extent, this shows agglomeration of particles in the composites. On the other hand, the 20 wt % TES filled composites showed an increase cross-link density value which is in consistent with *E*′ results.

The *tan δ* plot of pure epoxy, UES and TES particles filled epoxy composites with respect to temperature are shown in Figure 8B. The neat epoxy showed increased *tan δ* height value as compared to UES and TES particles filled composites; this represents the weak interfaces and frictional damping behavior. The deformation energy is dissolute mostly at the interface of composite materials. If the particle amount, geometry and matrix are identical, the *tan δ* value can be used to study the interfacial properties of composites. The higher *tan δ* height means the composite has a tendency to dissipate more energy—i.e., poor interfacial adhesion results in higher damping values. Moreover, the broadening of *tan δ* is related to molecular relaxations at the interfacial region. The introduction of ES particles reduces the height and increase the broadening of *tan δ* values, the composite showed increase of molecular interaction and relaxations at the interfacial regions. The smallest and broadening damping curves were observed for TES particles filled composites as compared to the UES particles filled composites [43]. This confirmed that TES particles filled composites have higher load bearing capacity than the UES particles filled composites. Moreover, a positive shift in loss modulus values to higher temperature of TES filled composites composite also represents more energy retention in contrast to UES filled composites and pure epoxy as shown in Figure 8C, which is in consistent with the *tan δ* results.

The effectiveness coefficient (*C*) was also calculated to cross check the adhesion effectiveness of particles and matrix in the composites. It is the ratio of composite storage modulus (*E*′) in glassy and rubbery region in relation to the neat resin, and calculated by using the equation [44]

(6) $$C=\frac{{E^{\prime }}_{g}/{E^{\prime }}_{r}\;composite}{{E^{\prime }}_{g}/{E^{\prime }}_{r}\;resin}$$

Here *E*′ represents storage modulus, while subscripts *g* and *r* represent glassy and rubbery regions, respectively.

The *C* for UES and TES particles filled composites was calculated at three different temperatures—i.e., 100, 120, and 140 °C—and values are shown in Table 5. The glassy region of composites and neat matrix was taken at 40 °C. The lower value of *C* represents more effectiveness for particle–matrix adhesion [45]. From the calculated values for *C*, we can conclude that the TES particles filled composite show lower values at all temperatures, which also cross verified the improved particle–matrix adhesion for TES particles than the UES particles.

#### 3.2.3. Tensile Properties

**Figure 9:**
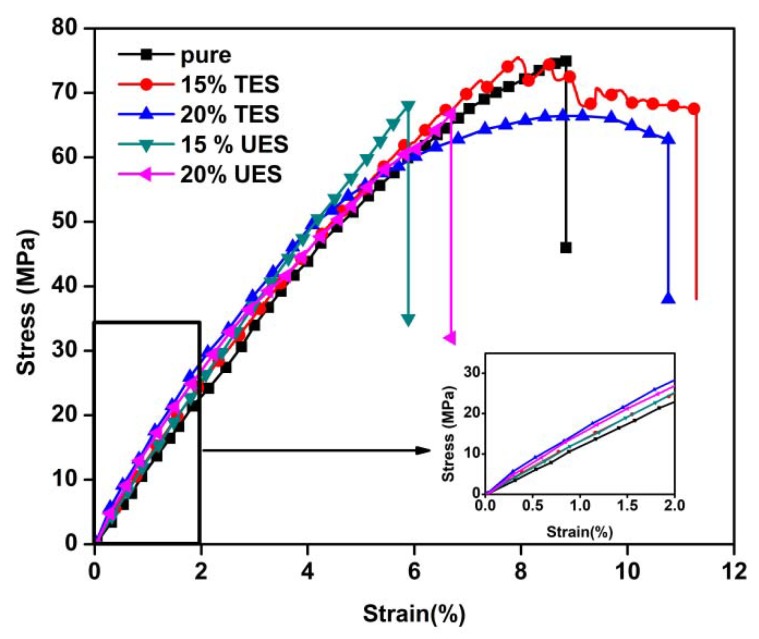
Tensile stress–strain curve of UES and TES filled epoxy composites on various compositions.

The tensile stress–strain behavior of epoxy filled particle composites is shown in Figure 9 and the corresponding values are tabulated in Table 6.

The UES particles filled epoxy composites showed semi ductile behavior, while TES particles filled epoxy composites showed increased ductility. It has been observed that on the macro size particles reinforcement ultimate strength of the composites was lower than the neat matrix [46]. On the other hand, the effective surface modification can change the behavior depending upon the bonding and size alteration. The tensile strength values at 15 and 20 wt % were recorded as 68.31 ± 1.95 and 66.44 ± 1.84 MPa, respectively, while the elongation was recorded as 5.87 ± 0.09 and 6.67 ± 0.11% for 15 and 20 wt % UES particles loading, respectively, the values for neat epoxy were 74.91 ± 2.21 MPa and 8.84 ± 0.15%. The highest elongation value (11.31 ± 0.18%) was recorded for 15 wt % TES filled epoxy composite, on 20 wt % TES loading the value reduced and recorded as 10.76 ± 0.12%. This enhancement in the elongation values can be explained by the reduced amount of protein and increased carbonyl groups due to SA treatment, improved dispersion of TES particle, reduced crystal size and increased hydrogen bonding as earlier discussed in FTIR. This employs the reduced brittleness (*B*) of the epoxy composites, the *B* was calculated by using the equation [47].

(7) $$B=\frac{1}{\varepsilon \;E^{\prime }}$$

Here ε is elongation at break and *E*′ is storage modulus by DMA. The calculated *B* values, summarized in Table 6, shows that the loading of TES particles filled epoxy composites have very lower brittleness as compared to UES particles filled epoxy composites and pure epoxy. These results are consistent with the toughness value, which confirms that TES filled epoxy composites have increased load bearing capacity with improved bending.

#### 3.2.4. TGA

**Figure 10:**
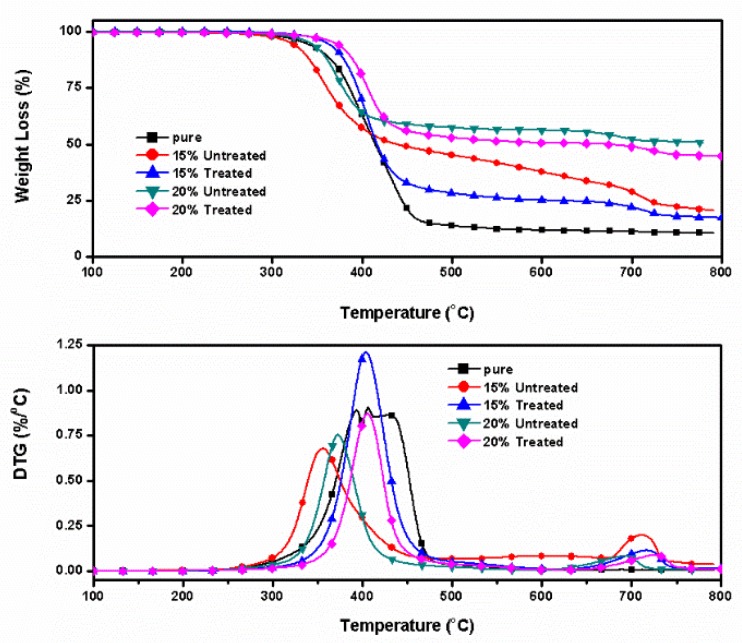
TGA and DTG curves of UES and TES filled epoxy composites on various compositions.

Thermal stability of UES and TES particles filled epoxy composites, and neat epoxy were evaluated in an inert atmosphere and illustrated as Figure 10. The calculated data for the degradation temperatures, including *T*_5%_, *T*_10%_, and *T*_20%_ weight loss temperatures, residual mass, and information on the maximum degradation intensity from DTG curves for UES and TES particles filled epoxy composites are summarized in Table 7.

The initial degradation temperature (values of *T*_5%_, *T*_10%_, and *T*_20%_) for 15 wt % UES particles containing epoxy composites were much lower than the neat matrix; however, the 20 wt % UES particles epoxy composites showed a slightly higher values, which may be due to the improved cross links formed between proteins and epoxy as discussed in DMA section. These results are much different than the reported literature due to the alkyl structure attached on the TES particles [18]. The UES particles only increases the char yield values for the composites which were much higher than the neat matrix. However, the TES particles also increase the thermal stability of composites. The TES particles filled epoxy composites also showed the improved initial degradation temperature as the loading of TES particles increased. The DTG peak values for the TES/epoxy composites were moved to higher temperature.

## 4. Conclusions

In the present work, the ES particles surface was modified with SA. The process was optimized on the basis of sediment volume, oil-wet coefficient, active ratio %, and viscosity ratio. The optimized process conditions were 2.5% concentration, 85 °C temperature, and 50 min treatment time. Afterwards, the UES and TES particles were characterized by conventional methods. The FTIR confirmed the attachment of SA on the particles surface, no phase transformation of calcite phase and reduced particle size were observed by XRD. The DSC showed the chemical bonding of SA to ES particles which increases the hydrophobicity of TES particles by increasing the contact angle to 169°. The morphological studies confirmed the removal of bio-impurities and formation of small particles. Later, the UES and TES particles were reinforced in the epoxy matrix at 15 and 20 wt %, and prepared composites were tested for FTIR, DMA, TGA, and tensile properties. The TES particles filled composites showed increased thermal stabilities, improved stiffness and higher *T_g_* as compared to the neat matrix. The toughness value from the tensile stress–strain plot showed a remarkable increase of 42 and 22% for 15 and 20 wt % TES composites and elongation increase to 27 and 16%, respectively. Moreover, the loading of TES also reduced the brittleness. The effectiveness of particle adhesion was also verified by calculating effectiveness coefficient (C), the TES filled composites showed lowest C value in comparison to the UES filled composites, which proved that the TES particles have increased particle–matrix adhesion. The new simple and economical modification technique for the modification of ES particles can be useful in the dispersion of ES particles in functional polymers, coating applications, and various other high-performance polymers for improved performance characteristics of structure at comparatively high loading levels.

## Figures and Tables

**Table 1:** Chemical composition of ES particles [9].

Composition	Amount (%)
Calcium carbonate	98.2
Magnesium	≈0.9
Phosphorous	≈0.9
Carbon, zinc, manganese, iron, and copper	<0.1

**Table 2:** Process optimization of SA treatment of ES particles.

SA Concentration (%)	Treatment Temperature (°C)	Treatment Time (min)	*V_set_* (mL/g)	*O_w_* (%)	*A_d_* (%)	*V_R_*
0	70	40	0.590	0.223	2.3	32.1
1			0.699	0.242	65.4	1.71
1.5			0.892	0.310	85.7	1.68
2			0.990	0.473	94.1	1.64
2.5			1.186	0.456	96.3	1.58
3			1.201	0.468	96.2	1.59
2.5	25	40	0.317	0.295	92.3	1.72
	45		0.332	0.285	94.2	1.63
	65		0.425	0.305	96.1	1.60
	85		0.428	0.468	96.3	1.58
	105		0.425	0.451	96.2	1.59
2.5	85	20	0.291	0.447	91.9	1.67
		30	0.376	0.452	92.1	1.63
		40	0.428	0.468	95.9	1.58
		50	1.178	0.472	96.3	1.58
		60	1.189	0.450	96.2	1.57

**Table 3:** XRD crystallite size parameters of UES and TES particles.

Sample	Dominant Peak 2*θ*	cos *θ* (rad)	Root Mean Square	*β*	*L* (nm)
UES	29.41	0.016888	0.979	0.17131	47.9
TES	29.46	0.016886	0.964	0.21113	38.9

**Table 4:** Particle size and polydispersity index (PDI) of aqueous dispersion of UES and TES.

Sample	Mean Particle Size (nm)	PDI
UES	1521	0.43
TES	683	0.39

**Table 5:** DMA data and effectiveness coefficient *C* of neat epoxy; UES and TES filled epoxy composite.

Sample	Storage Modulus (MPa)	Loss Modulus Peak (°C)	*tan δ* Peak Height	*tan δ* Peak (°C)	Cross-Link Density (10^−3^ mol/cm^3^)	*C*		
						100	120	140
Pure	1822	99	0.94	111	1.29	-	-	-
15% UES	2051	98	0.78	109	2.14	0.97	0.63	0.55
15% TES	2254	101	0.58	113	2.63	0.73	0.49	0.42
20% UES	2325	97	0.60	110	1.51	0.95	0.62	0.54
20% TES	2578	101	0.44	114	2.71	0.69	0.31	0.25

**Table 6:** Tensile and toughness values of composites.

Sample	Tensile Strength (MPa)	Elongation (%)	Toughness ^1^	Brittleness (%Pa × 10^−5^)
Pure epoxy	74.91 ± 2.21	8.84 ± 0.15	405 ± 4.10	5.5
15% UES	68.31 ± 1.95	5.87 ± 0.09	211 ± 2.69	9.29
20% UES	66.44 ± 1.84	6.67 ± 0.11	207 ± 2.91	11.4
15% TES	67.97 ± 2.11	11.31 ± 0.18	575 ± 5.10	3.79
20% TES	63.12 ± 2.07	10.76 ± 0.12	491 ± 3.87	3.84

^1^ area under stress strain curve value calculated by integration.

**Table 7:** TGA and DTG data of pure, UES and TES composites investigated under nitrogen atmosphere.

Sample	*T*_5%_ (°C)	*T*_10%_ (°C)	*T*_20%_ (°C)	Residual Mass (%)	DTG (°C; %/°C)
Pure epoxy	337	359	379	10.4	394; 0.89
15% UES	321	337	353	20.5	356; 0.68
15% TES	361	375	390	17.1	403; 1.21
20% UES	342	357	372	50.8	372; 0.75
20% TES	370	385	400	44.7	405; 0.87
